# Frequency multiplexed coherent φ-OTDR

**DOI:** 10.1038/s41598-021-97647-z

**Published:** 2021-09-09

**Authors:** Hannah M. Ogden, Matthew J. Murray, Joseph B. Murray, Clay Kirkendall, Brandon Redding

**Affiliations:** grid.89170.37 0000 0004 0591 0193 U.S. Naval Research Laboratory, 4555 Overlook Ave., SW, Washington, DC, 20375 USA

**Keywords:** Imaging and sensing, Physics

## Abstract

We present a comprehensive analysis of a frequency multiplexed phase-measuring φ-OTDR sensor platform. The system uses a train of frequency-shifted pulses to increase the average power injected into the fiber and provide a diversity of uncorrelated Rayleigh backscattering measurements. Through a combination of simulations, numerical analysis, and experimental measurements, we show that this approach not only enables lower noise and mitigates interference fading, but also improves the sensor linearity. We investigate the sensor dependence on the length of the pulse train and characterize the sensor performance as a function of range, demonstrating operation from 1 to 50 km. Despite its relative simplicity, this platform enables state-of-the-art performance, including low crosstalk, high linearity, and a minimum detectable strain of only 0.6 *p*$\varepsilon /\sqrt{\text{Hz}}$ in a 10 km fiber with 10 m spatial resolution and a bandwidth of 5 kHz.

## Introduction

Distributed acoustic sensors (DAS) based on Rayleigh scattering are capable of measuring dynamic strain events over long distances using low cost, off the shelf fiber. This combination makes these systems attractive for applications such as intrusion detection^1–3^, pipeline monitoring^4, 5^, and seismic activity detection^6–8^. This initial success has prompted an increasing demand for lower noise and longer range Rayleigh-based DAS. To that end, this work presents a systematic investigation of how frequency multiplexing can be used to improve the linearity, range, and sensitivity of Rayleigh-based DAS.

One of the most successful Rayleigh-based fiber sensing modalities is phase-sensitive optical time-domain reflectometry (φ-OTDR). These systems have advanced considerably over the past 20 years. Many of the first φ-OTDR systems simply measured the amplitude of the Rayleigh backscattered (RBS) light from a single pulse^1, 9^. While these systems were able to identify the presence of strain, the non-linear response of the RBS amplitude precluded quantitative measurements. The next advancement was the introduction of phase-measuring φ-OTDR systems^10–12^. These systems provided quantitative strain measurements by monitoring the relative phase between RBS light from two regions of the fiber. However, the requirement for coherent detection and susceptibility to interference fading^13^ inspired researchers to revisit amplitude measuring φ-OTDR systems. While the RBS amplitude at a single wavelength has a non-linear strain response, researchers found that quantitative sensing was possible by measuring the RBS amplitude at multiple wavelengths or spatial modes. This work led to the development of wavelength scanning φ-OTDR^14–16^, chirped pulse φ-OTDR^17–19^, and speckle tracking φ-OTDR^20, 21^. More recently, the focus has turned to developing techniques that also break the trade-off between spatial resolution and pulse duration which limits the optical launch power and, thus, the sensitivity of most φ-OTDR systems. These systems rely on frequency multiplexing to inject multiple pulses into the fiber at once^22^, or utilize modulated or chirped waveforms which can be “compressed” to achieve higher spatial resolution than the pulse duration would dictate^23–26^.

While these advances have led to some impressive demonstrations, many of the quantitative amplitude measuring schemes are computationally intensive^18^ and can be susceptible to large errors^27^. Moreover, chirped and modulated waveforms often require high bandwidth, high-fidelity arbitrary waveform generators, while techniques that break the trade-off between pulse duration and spatial resolution can be susceptible to crosstalk^25, 28, 29^. These complexities could make it challenging to transition some of these new designs from laboratory demonstrations to fielded systems with real-time operation without compromising performance. In this work, we investigate the potential for a frequency-multiplexed phase measuring φ-OTDR system to compete with these more recent innovations.

Although a number of frequency-multiplexed φ-OTDR systems have been presented in the literature, the ability for this approach to improve the fundamental noise performance and linearity has not been fully explored. In the past, frequency multiplexing was introduced in phase-measuring φ-OTDR systems as a means to address interference fading^30^ or to increase the sensor bandwidth^28, 31–33^. Frequency multiplexing has also been suggested as a potential method to improve the linearity of a phase-measuring φ-OTDR system^34^, although we are not aware of any work that experimentally investigated this dependence using more than 3 frequencies. In addition, some of these frequency-multiplexed studies reported counter-intuitive findings. For example, Ref.^32^ found that the signal-to-noise ratio (SNR) actually decreased if the system injected more pulses into the fiber. This trend was attributed to interference fading and crosstalk between frequency multiplexed channels, rather than an inherent limitation of the approach. Other researchers predicted a modest (though not necessarily monotonic) SNR improvement as the degree of multiplexing increased in a simulation, but only tested 3 frequencies experimentally^30^. Most of these demonstrations also remained susceptible to polarization fading, limiting the sensor performance. Finally, none of these works quantitatively characterized the system sensitivity in terms of the power spectral density of the phase or strain noise, as required to directly compare the performance with other φ-OTDR architectures.

We conducted a systematic study of a frequency-multiplexed phase measuring φ-OTDR system to probe the fundamental capabilities of this approach. The sensor uses a frequency shifted pulse train to increase the average power injected into the fiber and a weighted averaging approach to combine the information from each of these pulses. We present a combination of simulations, analytic noise performance analysis, and experiments to investigate the capabilities of this sensor platform. We experimentally characterized the spatial resolution, linearity, crosstalk, and noise spectral density to enable direct comparisons with competing approaches. We found that increasing the number of pulses injected into the fiber consistently improved both the sensor linearity and strain noise, providing a 13 dB noise reduction as we increased the number of pulses injected into the fiber from 1 to 20—matching the predicted performance improvement. This approach enables a high-performance φ-OTDR sensor capable of performing quantitative strain measurements with a total harmonic distortion of − 40 dB, crosstalk below − 40 dB and a minimum detectable strain of 0.6 $p\varepsilon /\sqrt{\text{Hz}}$ along 10 km of fiber with 10 m spatial resolution. We also investigate the range dependence and present measurements using fiber lengths from 1 to 50 km, achieving minimum detectable strains ranging from 0.1 to 9 $p\varepsilon /\sqrt{\text{Hz}}$. This combination of ultra-low noise with computationally simple processing makes this an attractive platform for next-generation, high-performance DAS.

### Operating principle

**Figure 1:**
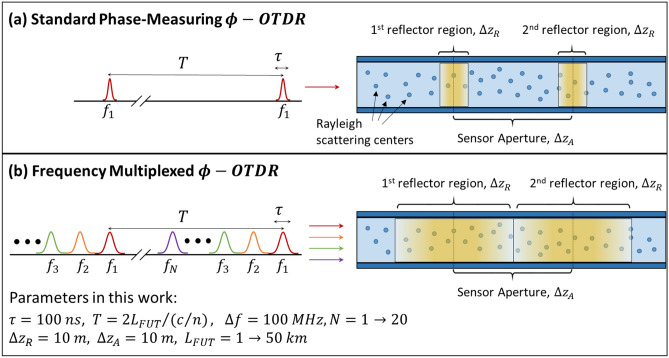
Schematic of operating principles of a standard (**a**) and frequency-multiplexed (**b**) phase measuring φ-OTDR system. The sensors are designed to measure the strain over a given sensor aperture by recording changes in the relative phase between RBS light from nearby reflector regions. Using frequency multiplexing, a train of *N* pulses can be injected into the fiber at once. This approach also enables longer pulses without compromising the sensor linearity—the reflector region size can be set equal to the desired sensor aperture size.

Phase measuring φ-OTDR sensors are designed to measure the optical phase accumulated over different sections of the fiber, since this phase will change in proportion to the strain in the fiber^35^. In practice, this is achieved by injecting a series of optical pulses of duration $\tau$ with a repetition period $T$, which is at least equal to the round-trip time in the fiber: $T\ge 2{L}_{FUT}/\left(c/n\right)$, where ${L}_{FUT}$ is the fiber length, $c$ is the speed of light, and $n$ is the effective index in the fiber. The sensor is then designed to measure the phase of the RBS light as a function of time, which can be converted to position in the fiber. The strain experienced by a section of fiber is obtained by monitoring the relative phase between RBS light from pairs of “reflector regions”, as shown in Fig. 1a. The size of these “reflector regions” is dictated by the pulse duration and given as $\Delta {z}_{R}=\left(\tau /2\right)\cdot \left(c/n\right)$. The sensor aperture size, $\Delta {z}_{A}$, is then defined by the separation between these reflector regions and the system will provide a linear strain response as long as the strain is confined to the region of fiber between these reflector regions. In practice, the aperture size is generally set to the spatial resolution required for a given application. The reflector regions can then be any size up to the aperture size without compromising this spatial resolution. However, if the reflector regions themselves are under strain, a linear response is no longer guaranteed^36, 37^. This non-linear response results from a strain-induced change in the relative spacing between Rayleigh scattering centers in the reflector region, which can introduce an unpredictable change in the RBS phase (and amplitude). This introduces a trade-off in standard phase measuring φ-OTDR systems between sensitivity and linearity. A sensor that uses larger reflector regions (i.e. longer pulses) will be able to inject higher average power into the fiber resulting in lower noise. At the same time, the non-linear response of the reflector regions themselves will have a greater impact as their length approaches the aperture size, reducing the overall sensor linearity^34, 38^.

The frequency multiplexed phase-measuring φ-OTDR system presented in this work enables significantly lower noise by addressing this trade-off while also increasing the effective pulse repetition rate. Instead of launching a single pulse with a repetition period $T$, the proposed system injects a train of $N$ pulses with different optical frequencies every period. Since the modulation instability threshold typically limits the peak power that can be injected into the fiber^39^, using a pulse train enables the system to increase the average power coupled into the fiber by a factor of *N*, as shown in Fig. 1b. In addition, we will show that averaging the response from many uncorrelated frequencies allows us to maintain a linear strain response even when the reflector region size is matched to the aperture size (i.e. $\Delta {z}_{R}=\Delta {z}_{A}$). This approach to improving the sensor linearity through multiplexing was discussed in Ref.^34^, but to our knowledge, this approach has not been investigated experimentally using more than 3 frequencies^30^. Matching the size of the reflector region to the aperture size allows us to use the maximum pulse duration allowed for a desired spatial resolution (without resorting to pulse compression techniques). By using longer pulses and increasing the number of pulses per repetition period, the frequency multiplexed φ-OTDR system has the potential to achieve dramatically lower noise than standard phase measuring φ-OTDR systems.

We also show that the frequency-multiplexed approach can efficiently suppress interference fading. Interference fading occurs when the amplitude of the Rayleigh backscattered light approaches zero due to destructive interference. In a standard phase-measuring φ-OTDR system, this results in significantly higher phase noise at seemingly random positions in the fiber where the RBS amplitude is low. Fortunately, these fading positions depend on the optical frequency^40^. By increasing the number of probe frequencies, we can quickly reduce the probability that each frequency will suffer interference fading at the same position, ensuring that we obtain at least some high quality measurements at every position in the fiber^30^. In this work, we use an amplitude-weighted averaging approach to combine the *N* measurements, efficiently reducing the impact of measurements with low-amplitude and higher phase noise. As a result, the frequency-multiplexed φ-OTDR sensor provides a low and uniform phase noise across the entire fiber.

### Simulated sensor performance

**Figure 2:**
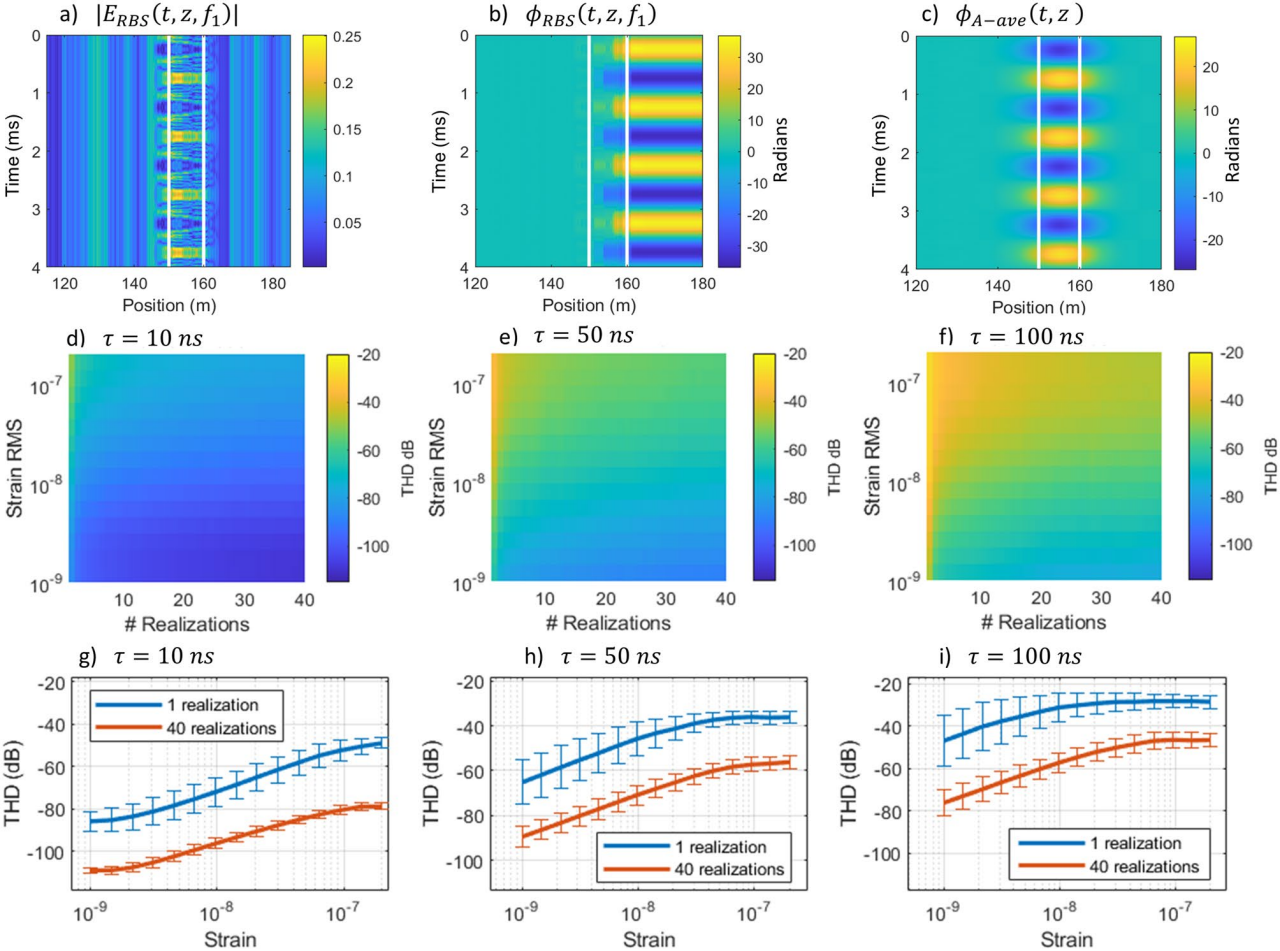
Results from simulations based on Rayleigh backscattered light from a 1 km FUT probed with a multi-frequency pulse train. The simulated amplitude (**a**) and phase (**b**) of an individual frequency component as a function of position along the fiber. (**c**) The aperture phase averaged over 40 frequency components. THD as a function of strain amplitude and number of realizations for 10 (**d**), 50 (**e**), and 100 (**f**) ns. Cross-sections of the THD for 1 and 40 realizations as a function of the strain amplitude at 10 (**g**), 50 (**h**), and 100 (**i**) ns. Error bars are calculated from 60 repetitions of the simulation for each set of parameters.

We first conducted a series of simulations to evaluate the sensor linearity as a function of the strain experienced by the fiber, the number of pulses in the pulse train, and the reflector region size (relative to a fixed aperture size). We used a standard impulse response model to simulate the RBS field^41^ as a function of the optical launch frequency and introduced strain to the simulated fiber by adjusting the position of the scattering centers. We calculated the Rayleigh backscattered field for each launch frequency, $f$, and at each time, $t$, as:

1 $$E\left(f,t\right)=\sum_{n=1}^{N}{r}_{n}\text{exp}\left[i2\pi \nu t+i2{\beta }_{f}\left({z}_{n}+\xi \varepsilon \left(t\right){z}_{n}\right)\right]rect\left(\frac{t-{t}_{d}}{\uptau }\right),$$

where ${r}_{n}$ is the reflectivity of the *n*th scattering center, ${\beta }_{f}$ is the propagation constant at launch frequency, $f$, ${z}_{n}$ is the position of the *n*th scatterer, $\varepsilon$ is the strain, $\xi$ is the elasto-optic coefficient, and ${t}_{d}$ is the temporal delay to the sensor region^21, 41^. The simulation provided the RBS amplitude, $\left|{E}_{RBS}\right|$, and phase, ${\phi }_{RBS}$, as a function of position in the fiber over time (as we varied the strain in the fiber) as shown in Fig. 2a,b. In this simulation, a sinusoidal strain was introduced with a frequency of 1 kHz extending from 150 to 160 m into the simulated fiber (the strained region is indicated by white lines in Fig. 2a–c). The non-linear response of the RBS amplitude to strain is clearly visible in Fig. 2a. We obtained this type of simulated RBS field for 40 launch frequencies in steps of 100 MHz starting at a nominal laser frequency of 193.5 THz. This frequency separation was sufficient to ensure that the RBS fields were uncorrelated^40^. We then used a weighted averaging technique to combine the response of each frequency (similar to the rotated vector sum technique reported in Refs.^26, 42^). To perform the weighted averaging, we first calculated the phase accumulated over a given sensor aperture at each time, $t$, position, $z$, and launch frequency, $f$, as:

2 $${\phi }_{A}\left(t,z,f\right)={\phi }_{RBS}\left(t,z,f\right)-{\phi }_{RBS}\left(t,z+\Delta {z}_{A},f\right).$$

We then performed a weighted average using the temporal derivative of the phase at each position:

3 $${\Delta \phi }_{A}\left(t,z,f\right)={\phi }_{A}\left(t,z,f\right)-{\phi }_{A}\left(t+T,z,f\right),$$

4 $${\Delta \phi }_{A-ave}\left(t,z\right)=\frac{\sum {\Delta \phi }_{A}\left(t,z,f\right)\cdot \left|{E}_{RBS}\left(t,z,f\right)\right|}{\sum \left|{E}_{RBS}\left(t,z,f\right)\right|}.$$

The summations in Eq. (4) were performed over frequency and ${\Delta \phi }_{A-ave}$ describes the average phase change across a given sensor aperture during the time between measurements (i.e. at the pulse repetition period). Finally, the temporal evolution of the phase across each aperture, ${\phi }_{A-ave}(t,z)$, was obtained by integrating ${\Delta \phi }_{A-ave}$ at each position in the fiber, as shown in Fig. 2c. This phase evolution is proportional to the strain experienced over a given sensor aperture: ${\varepsilon }_{A}\left(t,z\right)={\phi }_{A-ave}\left(t,z\right)\cdot \lambda /(4\pi n\xi\Delta {z}_{A})$^35^. Finally, we calculated the power spectral density (PSD) of the strain at the center of the strained section of fiber (at 155 m in our simulation) in order to calculate the total harmonic distortion (THD) of the recovered strain response. The THD (in dB) is defined as $THD=20{{\text{log}}_{10}[ ({\varepsilon }_{{2\cdot f}_{sig}}^{2}+{\varepsilon }_{{3\cdot f}_{sig}}^{2}\dots +{\varepsilon }_{{10\cdot f}_{sig}}^{2})}^{1/2}/{\varepsilon }_{{f}_{sig}}]$ , where ${\varepsilon }_{{f}_{sig}}$ is the strain at the signal frequency.

We repeated this process, calculating the THD while varying the launch pulse duration from 10 to 100 ns and keeping the aperture size fixed at 10 m (typical of many $\varphi$-OTDR sensors). We also adjusted the strain in the fiber from 1 to 200 nε and calculated the weighted average over 1 to all 40 frequencies. This covers a common range of strain values encountered by many $\varphi$-OTDR systems, while 40 frequencies matched the maximum number of realizations our system was setup to probe experimentally (i.e. by using 20 pulses and recording 2 polarizations states). Finally, since the non-linear response of the reflector regions depends on the random distribution of scattering centers in the fiber, we repeated the simulation at each set of parameters using 60 random ensembles of Rayleigh scattering centers (defined by ${r}_{n}$ and ${z}_{n}$ in Eq. (1). The results of these simulations are summarized in Fig. 2d–i.

The simulations show that the THD increases with pulse duration due to an increase in the size of the reflector region relative to the aperture size. These simulations also show that the distortion initially increases with strain before saturating once the phase modulation introduced by the strain approaches $\pi .$ Finally, we found that averaging over an increasing number of frequencies consistently improves the sensor linearity. For example, in the simulations with $\tau$ set to 100 ns such that the reflector region size matched the aperture size, the THD approached − 20 dB for a single frequency. However, averaging over 40 realizations reduced the THD to below − 40 dB, sufficient for most applications. This indicates that the frequency multiplexing approach should enable the system to use longer pulses without compromising the sensor linearity.

## Methods

**Figure 3:**
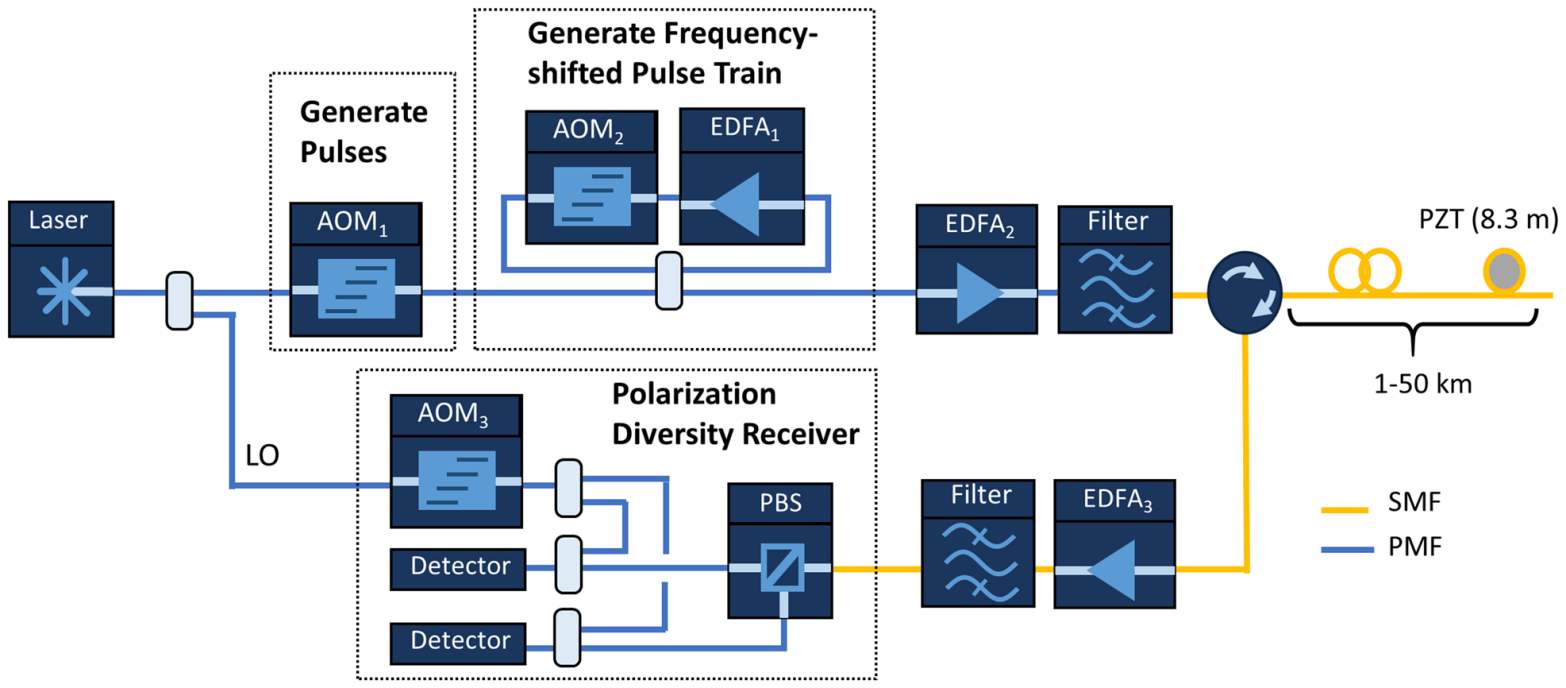
Experimental setup used to interrogate the fiber with a train of frequency shifted pulses. A single narrowband laser is divided into an interrogation and LO arm. AOM_1_ generates 100 ns pulses and introduces a frequency shift of 195 MHz. AOM_2_ controls the number of pulses in the pulse train and imparts a + 100 MHz frequency shift between pulses. AOM_3_ shifts the LO by − 55 MHz to offset the interference terms between the LO and the RBS light from the cross-terms formed by interference between RBS light from different pulses in the pulse train.

Based on the results of our simulation, we constructed a frequency multiplexed φ-OTDR system capable of generating a train of *N* frequency-shifted pulses. A schematic of the experimental setup is provided in Fig. 3. A single narrowband (< 5 Hz linewidth) continuous-wave laser operating at $\lambda =$ 1550 nm was divided into an interrogation arm and a reference or local oscillator (LO) arm. In the interrogation path, the first acousto-optic modulator (AOM_1_) was used to carve a 100 ns pulse at a repetition period set by the length of the FUT. In the experiments reported in this work, we fixed the pulse duration at 100 ns, corresponding to a 10 m reflector region. The sensing aperture was set to 10 m during data processing, although this could be adjusted computationally depending on the application. AOM_1_ also introduced a frequency shift of + 195 MHz. This type of recirculating loop enables the efficient generation of a train of pulses with equal frequency spacing without requiring high bandwidth modulation^22, 30, 43^. In this work, we evaluated the sensor performance using pulse trains of up to 20 pulses. The pulse train was then amplified using a second EDFA to a peak power of ~ 200 mW (below the modulation instability threshold^39^). A 100 GHz bandwidth wavelength division multiplexing filter was used to suppress amplified spontaneous emission (ASE) before the pulse train was coupled into the FUT. The RBS light was amplified with a final EDFA and then coupled into a polarization diversity receiver where it was mixed with the LO on a pair of high-speed (10 GHz) AC-coupled photodetectors and digitized at 5 GS/s.

The LO was shifted by − 55 MHz using AOM_3_, resulting in interference frequencies between the LO and the RBS light starting at 250 MHz and increasing in steps of 100 MHz for each pulse. This design ensured that the cross terms produced by the pulses interfering with each other (which started at 100 MHz and increased in steps of 100 MHz) did not overlap with the desired signals produced by interference between the LO and the RBS pulses.

While this approach requires coherent detection, the sensor is not as susceptible to laser phase noise as one might initially expect. Although the path mismatch between the RBS light and the LO approached 100 km (twice the FUT length) in some of our experiments, laser phase noise will have nearly the same effect on the demodulated phase from neighboring reflector regions. Thus, when we calculate the phase over a given aperture using Eq. (2), this common laser phase noise is suppressed. The system remains sensitive to laser phase noise on the time-scale of the pulse duration (100 ns), but does not require a coherence length in excess of the FUT length. In practice, our residual sensitivity to laser phase noise will depend on how accurately we are able to computationally remove correlated noise from two measurements of neighboring reflector regions.

After digitization, we used I/Q demodulation to recover the RBS field generated by each pulse. We then used the same processing outlined in the simulation section to extract the average response of the pulse train. Note that the RBS field recorded by each polarization is treated as an uncorrelated RBS realization in the same way as measurements at different optical frequencies.

## Results and discussion

We used the basic setup shown in Fig. 3 to conduct a series of experiments to evaluate the performance of the frequency multiplexed φ-OTDR platform. In particular, we evaluated the sensor strain noise and linearity while varying the number of pulses from 1 to 20. We also investigated the range dependence by probing fibers from 1 to 50 km in length. Finally, we characterized crosstalk in the sensor.

**Figure 4:**
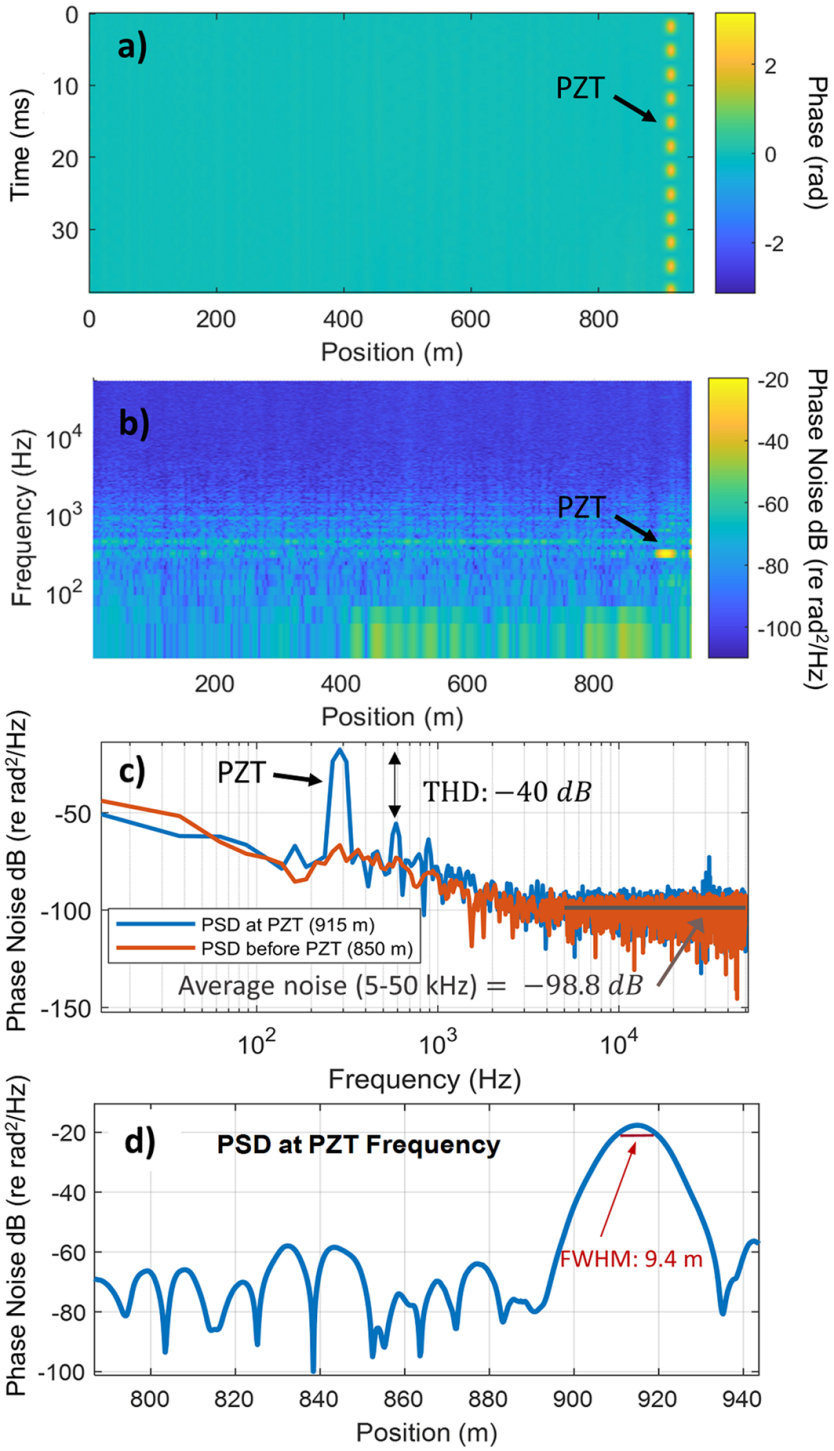
Typical measurement obtained using a train of 20 pulses and a 1 km FUT. (**a**) Temporal evolution of the phase at each position in the fiber, showing the modulation introduced by the PZT. (**b**) Spectrogram showing the PSD at each position in the fiber. (**c**) The phase PSD at the PZT position, confirming that the PZT modulation was measured with a high degree of linearity. The average phase noise (in the frequency range of 5–50 kHz) is shown as a solid horizontal line. (**d**) Cross-section of the PSD at the PZT frequency versus position. The PZT signal is measured at 915 m with a FWHM of 9.4 m indicated by the red solid line.

A typical measurement obtained using 40 realizations (20 pulses with 2 polarizations) and a 1 km FUT is shown in Fig. 4. In this case, a PZT wrapped with 8 m of fiber was positioned 915 m into the FUT and introduced a sinusoidal strain at 300 Hz. The PZT modulation is clearly visible in Fig. 4a, which shows the time-varying phase (which is proportional to strain) at each position in the fiber. A spectrogram of the strain at each position is shown in Fig. 4b, and a cross-section of the power spectral density (PSD) at the PZT position is shown in Fig. 4c. The PSD reveals that the PZT modulation was recovered with a high degree of linearity ($THD\sim -\,40 \text{dB}$). The full-width at half-maximum (FWHM) of the signal from the PZT was 9.4 m as shown in Fig. 4d, confirming that the spatial resolution matched the sensor aperture size.

In order to evaluate the sensor noise, we also plotted the phase noise PSD at a position before the PZT in Fig. 4c. The phase noise increased at low frequency due to environmental noise in the lab, but reached a flat noise level beyond ~ 3 kHz. To evaluate the self-noise of the sensor, we calculated the average phase noise from 5 to 50 kHz which was − 98.8 dB (re rad^2^/Hz), as indicated by the gray line on Fig. 4c.

This measurement also confirmed that interference and polarization fading were efficiently suppressed by performing a weighted average over 40 realizations. In particular, Fig. 4b does not show the large fluctuations in phase noise that are typical of a φ-OTDR system that is sensitive to fading. To quantitatively evaluate the phase noise fluctuation, we calculated the average phase noise from 5 to 50 kHz at each position in the fiber. We found that the phase noise had an average value of − 99.3 dB (re rad^2^/Hz) with a standard deviation of 0.8 dB across the FUT. The highest phase noise at any position in the fiber was − 97 dB (re rad^2^/Hz). This consistently low noise level is evidence that interference fading is not degrading the sensor performance.

### Sensor linearity

**Figure 5:**
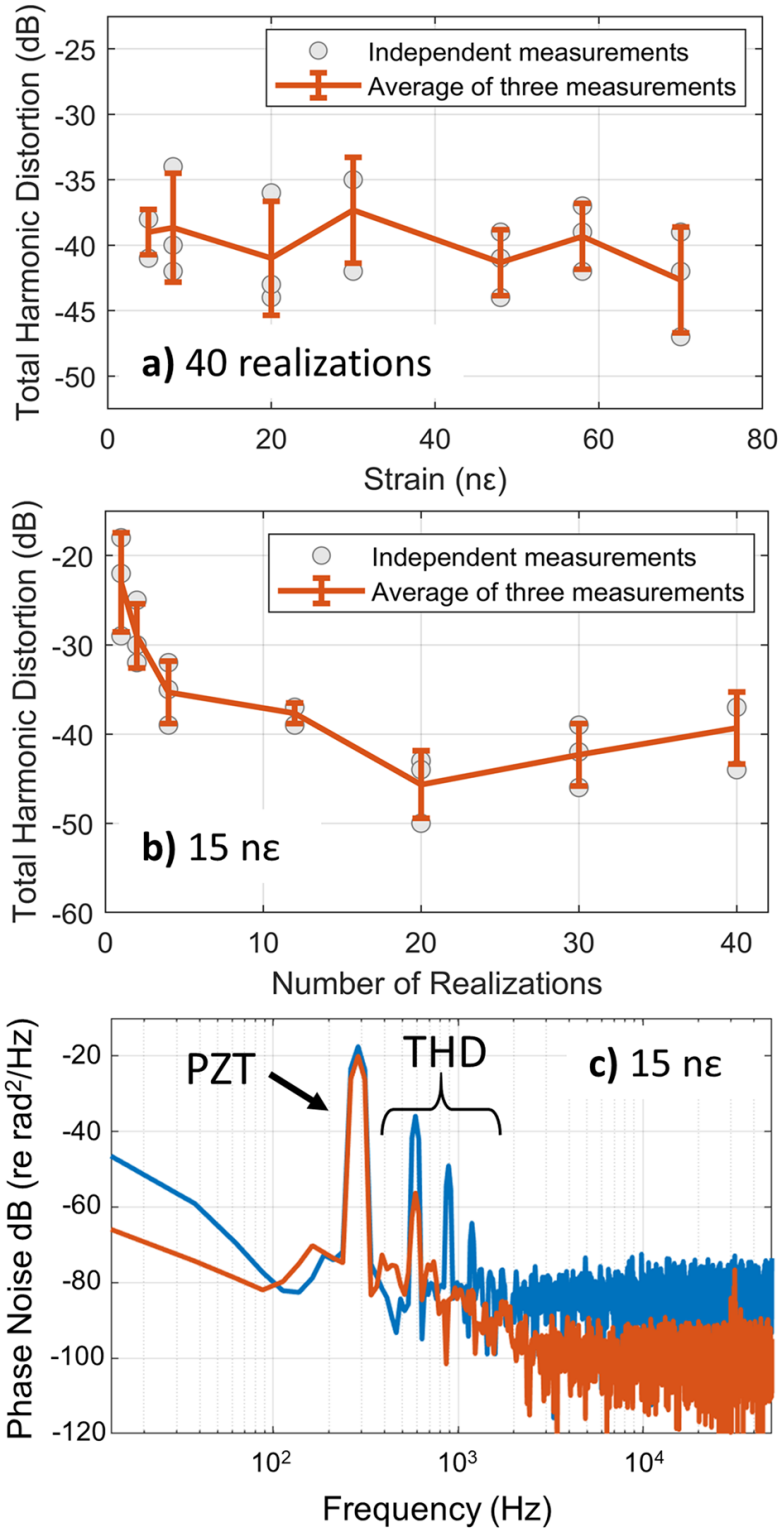
Analysis of the sensor linearity. (**a**) The total harmonic distortion at 5 distinct strain levels using 20 pulses (40 realizations). The measured THD remained below − 33 dB regardless of strain amplitude. The average of three independent experimental measurements are shown along with the standard deviation at each strain level. (**b**) The THD as a function of the number of realizations used to probe the FUT. The THD decreased rapidly as more realizations were used to obtain the average sensor response at a fixed strain amplitude of 15 $n\varepsilon$. (**c**) Example PSD obtained using 1 or 40 realizations with a 300 Hz strain. The THD was suppressed using 40 realizations.

To evaluate the sensor linearity, we recorded a series of measurements similar to that shown in Fig. 4 while varying the strain amplitude from 5 to 70 nε. The THD obtained after performing a weighted average over all 40 RBS realizations (20 pulses and 2 polarizations) is shown in Fig. 5a as a function of strain amplitude. For each of the 5 distinct strain amplitudes, the THD obtained from three experimental measurements are indicated with gray circles while the average and standard deviation are shown as a solid orange line. This measurement shows that the THD was consistently below − 33 dB, despite setting the reflector region size to match the aperture size ($\Delta {z}_{R}=\Delta {z}_{A}=10\, \text{m})$. We also note a THD below − 40 dB is limited by the noise floor of the sensor, particularly in the case of weak strain signals. As a result, we did not see a decrease in the distortion at the lowest strain amplitudes measured (as predicted by the simulations). To illustrate the importance of averaging over many realizations, we also calculated the THD while varying the number of realizations used in the weighted average. As shown in Fig. 5b, the THD was as high − 18 dB when a single realization was used (as in standard phase-measuring φ-OTDR systems), but decreased rapidly with an increasing number of realizations. At each “number of realizations” used to probe the FUT, we show 3 individual measurements (gray circles) and their average and standard deviation (solid orange line). Figure 5c shows example PSDs obtained using either 1 or 40 realizations, showing a distinct decrease in THD achieved through frequency multiplexing. This measurement confirmed that frequency multiplexing enables highly linear measurements even when the pulse duration is maximized for a desired spatial resolution.

### Sensor noise

**Figure 6:**
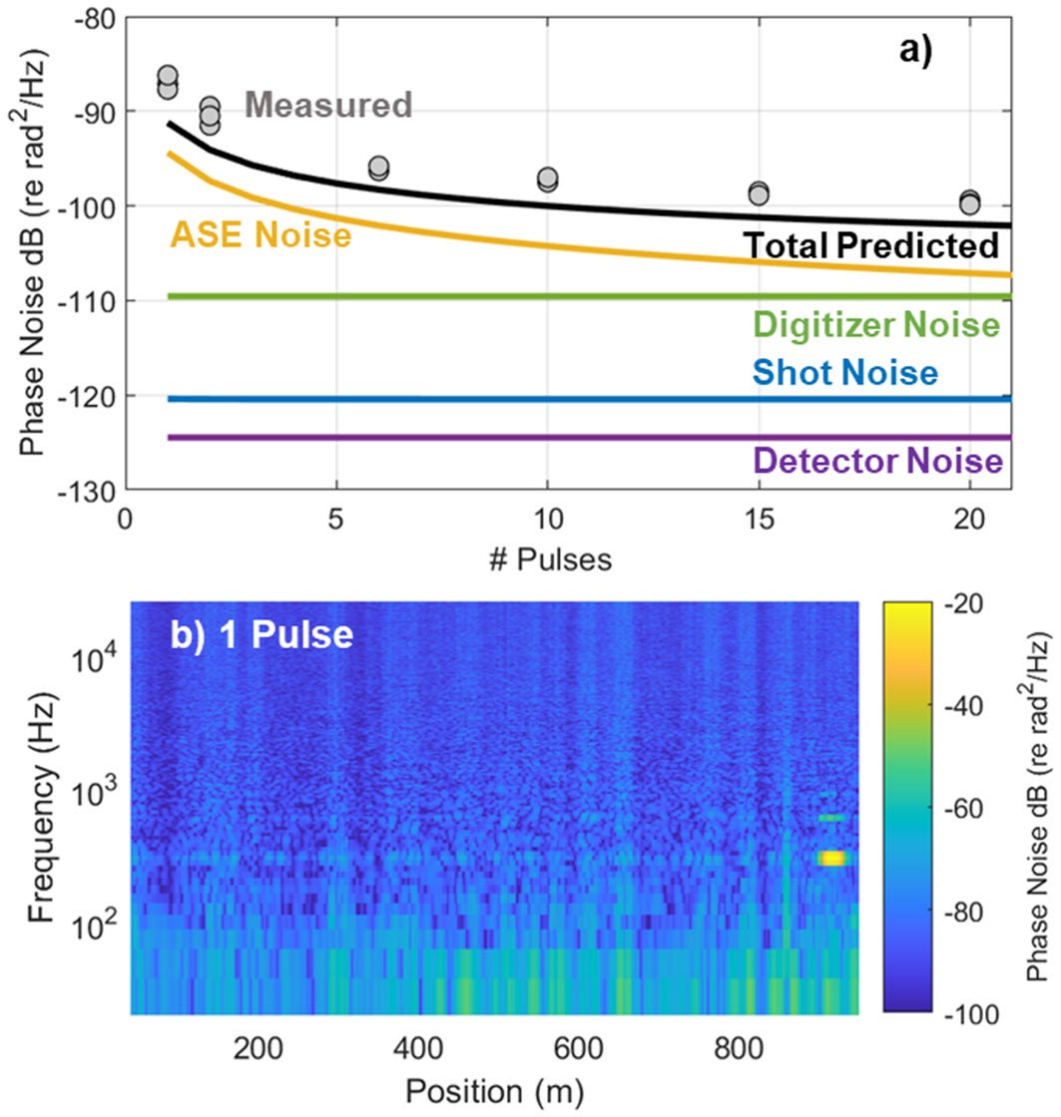
Pulse-dependent noise performance. (**a**) The predicted phase noise due to the dominant noise sources compared to the experimentally measured phase noise. (**b**) A typical spectrogram obtained using a single pulse. Note the variations in the phase noise due to interference fading and the harmonics of the PZT modulation signal at 300 Hz, indicative of a non-linear sensor response.

In order to optimize the sensitivity of the frequency multiplexed φ-OTDR sensor, we performed a numerical analysis of the dominant noise sources following the technique described in Refs.^35, 44^. This analysis considered noise introduced by ASE from each EDFA, optical shot noise, photodetector noise (based on the noise-equivalent-power of the detector), and noise introduced by the digitizer (based on the effective number of bits). Additional details for calculating the noise contributions from each of these sources are included in the Supplementary Information. We used this model to predict the contribution from each noise source as a function of the number of pulses in the pulse train. The predicted phase noise contributions for a 1 km FUT are shown in Fig. 6a, assuming a fixed peak launch power of 200 mW and 100 ns pulses. Note that the total predicted noise is 3 dB higher than the sum of the individual noise sources, since the phase noise over a given sensor aperture is obtained by calculating the relative phase between two reflector regions (i.e. in Eq. (3)).

This analysis indicated that the dominant noise source in our system is ASE introduced by the EDFAs. The ASE noise steadily decreases with the number of pulses, since the gain introduced by EDFA_3_ was adjusted in our model (and in our experiments) to maintain a constant average power on the photodetector—the optical power was kept just below the detector saturation level. By keeping the detected power constant, the photodetector, digitizer, and shot noise are independent of the number of pulses. Of course, if we decreased the EDFA gain to reduce the ASE noise, the remaining noise sources (digitizer, detector, and shot-noise) would increase and preclude us from achieving a lower overall noise. In other words, the EDFA helps to mitigate technical noise from the digitizer and photodetector and the predicted ASE noise shown in Fig. 6a represents a practical limit on the sensor performance for a given number of pulses.

We then compared this prediction with the experimentally measured phase noise obtained using a 1 km FUT. Experimentally, we recorded a series of measurements while adjusting the number of pulses in the pulse train and calculated the average phase noise in the frequency band from 25 to 50 kHz (selected to avoid the impact of environmental noise). We found that the experimentally measured noise, shown as gray circles in Fig. 6a, followed the predicted trend and agreed well with the analytic prediction for 6 or more pulses. The variation was slightly higher in the case of 1 or 2 pulses due to the impact of interference fading, which is not fully suppressed with so few pulses. This can also be seen in the spectrogram shown in Fig. 6b which was obtained using 1 pulse and shows significant variations in the phase noise as a function of position along the fiber. This is in contrast to the spectrogram shown in Fig. 4b, which was obtained using 20 pulses, and shows both a lower and more consistent phase noise level. The measurements summarized in Fig. 6a also confirmed that increasing the number of pulses from 1 to 20 provided the expected ~ 13 dB noise reduction due to the increased optical power injected into the FUT. The agreement with the predicted noise indicated that the experimental system was not limited by other factors, such as laser phase noise or frequency jitter introduced by the AOMs, which were not included in the model. Finally, this analysis indicates that increasing the number of pulses beyond 20 would provide diminishing returns in terms of noise performance, since the digitizer noise begins to dominate beyond ~ 30 pulses.

**Figure 7:**
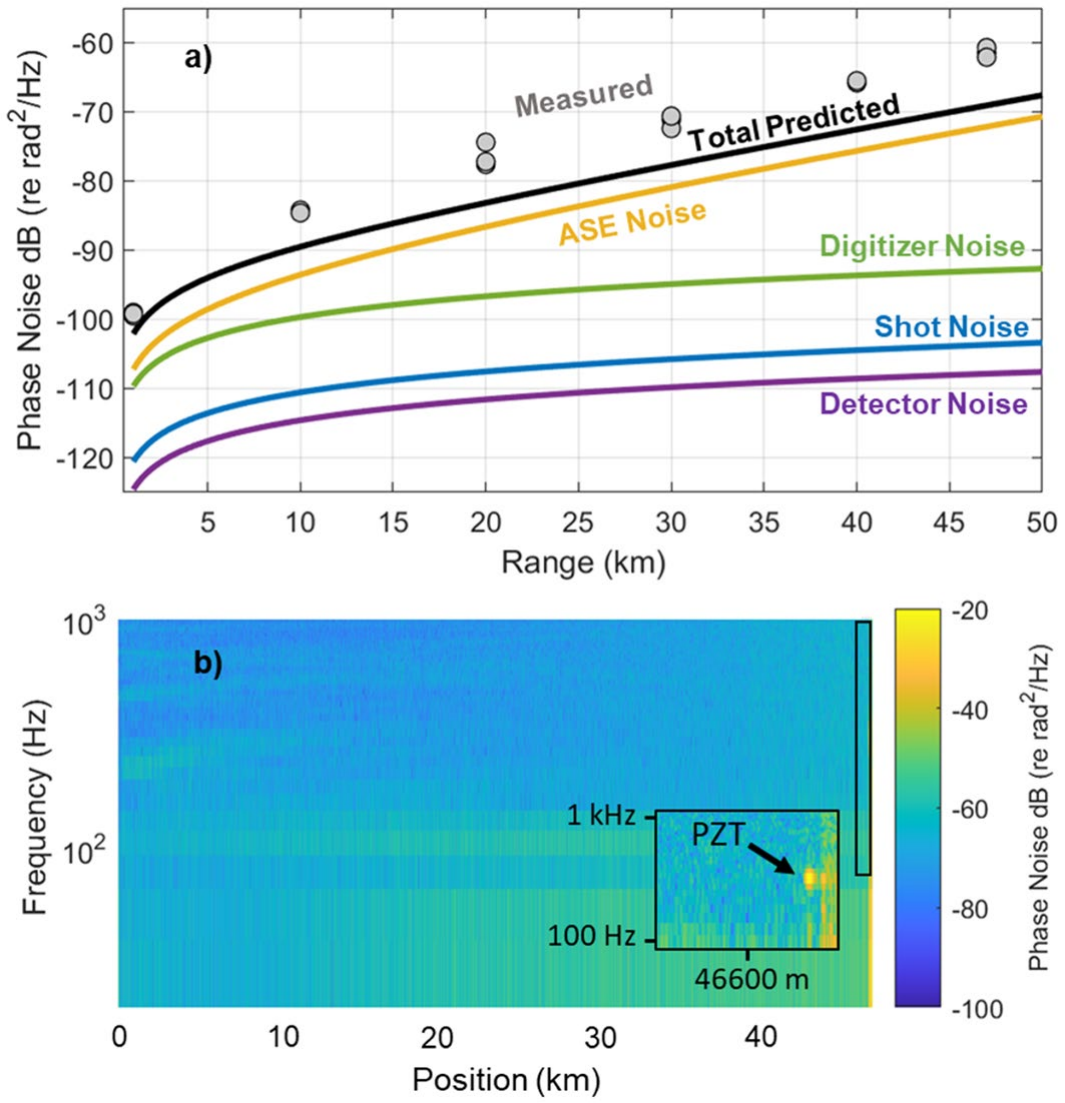
Range dependence of the sensor. (**a**) The predicted phase noise is shown as a function of the FUT length and compared to the experimentally measured noise. The measured noise follows the predicted trend, but diverges slightly at long range. (**b**) A typical spectrogram recorded using 20 pulses and a 47 km FUT. Inset: The PZT modulation is clearly visible at end of the FUT.

We then performed a series of experiments using fibers ranging from 1 to 47 km. In each case, we used a train of 20 pulses and fixed the peak launch power at 200 mW. The pulse repetition period was adjusted to match the round trip time in the FUT. A PZT was placed at the end of the FUT to ensure the system could successfully recover a dynamic strain signal at long range. We also used the phase noise model described above to calculate the expected sensor noise as a function of range. The predicted phase noise along with the measured phase noise at 6 different ranges are shown in Fig. 7. While the predicted and measured phase noise both increase with range (due to attenuation and the change in the pulse repetition period), we found that the experimentally measured phase noise diverges slightly from the prediction. In particular, while the measured noise is in excellent agreement at a range of 1 km, we found that the measured noise was as much as 7 dB higher than the predicted noise as we approached 50 km. One explanation is that laser phase noise could limit the sensor performance at longer ranges. After all, with a 50 km FUT the path mismatch can reach 100 km between the RBS light and the LO. This could introduce large phase variations which could be challenging to suppress computationally by calculating the relative phase between neighboring reflector regions. To investigate this possibility, an additional 10 km of fiber was added before AOM_3_ in the LO path labelled in Fig. 3. If laser phase noise were significant, we would have expected to see a dip in the measured phase noise 5 km into the FUT (where the path mismatch approaches zero). However, the measured phase noise was unchanged, indicating that laser phase noise is likely not a significant factor in the sensor performance.

Instead, we attribute the divergence between the measured phase noise and the predictions at long range to environmental noise in the laboratory. The environmental noise has an increased effect on the longer range measurements due to their reduced sensor bandwidth. For example, when the system was setup to probe a 50 km fiber, the repetition rate was reduced to 2 kHz, providing a sensor bandwidth of only 1 kHz. As can be seen in Fig. 4c, the environmental noise in the laboratory can extend to ~ 2 kHz. This environmental noise will then alias into the available bandwidth, obscuring our measurement of the sensor self-noise. In contrast, in the 1 km test, we were able to measure the sensor self-noise in the frequency band beyond ~ 5 kHz where environmental noise was negligible.

While it is beyond the scope of this work, we expect that the frequency multiplexing architecture could be extended to enhance the sensor bandwidth, similar to the approach presented in Ref.^28^. This would require us to adjust the timing of the pulse train to inject pulses into the fiber at constant intervals, rather than as a burst of closely spaced pulses. Experimentally, this could be achieved by adding a delay line in the recirculating loop to control the interval between pulses in the pulse train.

Despite the deviation from the predicted noise at ranges beyond 1 km, the frequency multiplexed φ-OTDR sensor reported here still provides exceptionally low noise. For example, at a range of 10 km, the measured phase noise of − 84.5 dB (re rad^2^/Hz) corresponds to a strain noise of just 0.6 $p\varepsilon /\sqrt{\text{Hz}}$. For comparison, two recent amplitude measuring φ-OTDR systems reported strain noise in the few $p\varepsilon /\sqrt{\text{Hz}}$ level for a 10 km FUT with the same ~ 10 m spatial resolution^18, 22^.

### Sensor crosstalk

**Figure 8:**
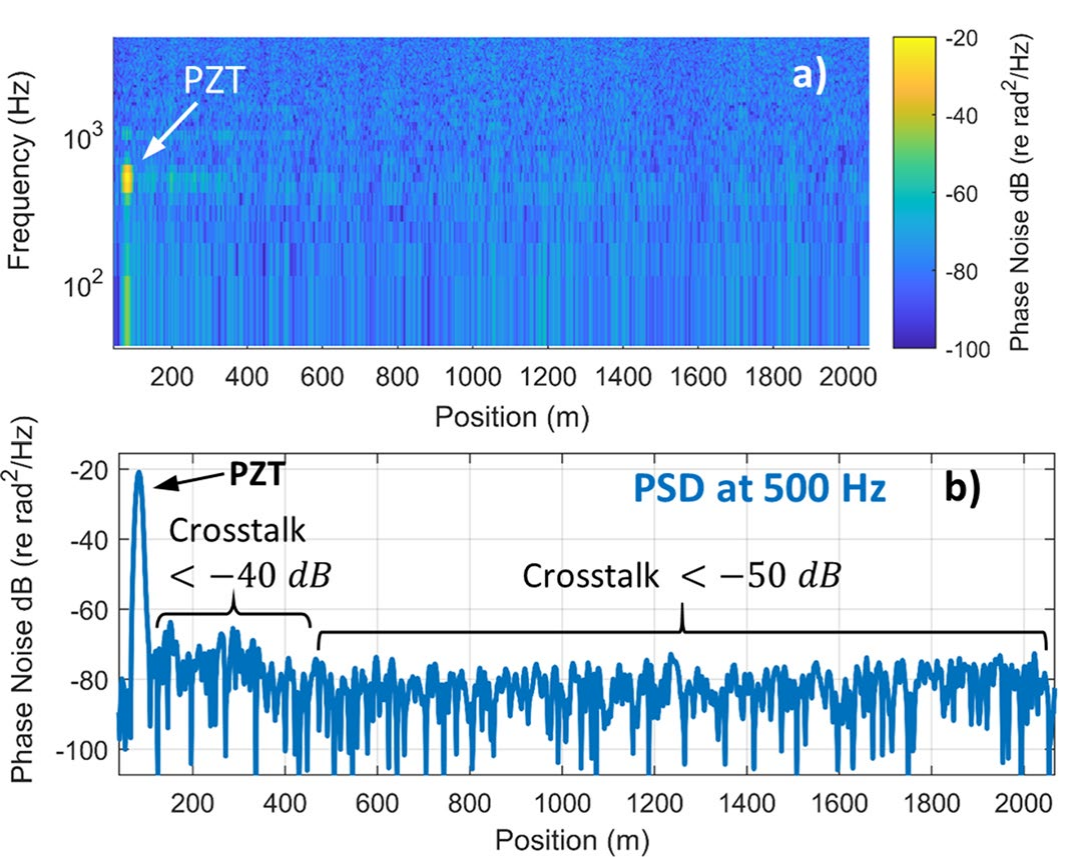
Crosstalk performance (**a**) Spectrogram showing the first 2100 m of a 10 km FUT with a PZT near the beginning of the fiber. (**b**) Cross-section of the PSD at 500 Hz as a function of position in the fiber. The PZT signal was suppressed by at least 40 dB in the first 400 m of fiber and by at least 50 dB in the rest of the FUT.

In distributed fiber sensors, crosstalk occurs when strain at one position in the fiber impacts the measured strain at other positions in the fiber. While standard, singe pulse φ-OTDR systems are relatively immune to crosstalk, emerging approaches that break the trade-off between pulse duration and spatial resolution can be more susceptible. For example, a recently reported frequency multiplexed amplitude-measuring φ-OTDR system exhibited crosstalk of − 20 dB within 50 m of a strain event^22^ while a system using linearly chirped pulses and non-matched filters observed crosstalk of − 23 dB^25^. To evaluate crosstalk in our system, we placed the PZT near the front of a 10 km FUT and introduced a relatively large 500 Hz signal with a strain amplitude of 15 $n\varepsilon$. The measured spectrogram acquired using a train of 20 pulses is shown in Fig. 8a. The PZT signal is clearly visible and some evidence of a 500 Hz signal is visible within the first 400 m of the fiber. To quantitatively evaluate the sensitivity of other positions to the applied PZT signal, we plotted a cross-section of the spectrogram at the 500 Hz signal frequency as a function of position, as shown in Fig. 8b. These figures reveal that crosstalk is suppressed by at least 40 dB within ~ 400 m of the strain event. Outside this distance, the residual PZT signal is below the sensor noise floor, indicating that the signal is suppressed by at least 50 dB. The slightly higher crosstalk near the strain event results from crosstalk in the frequency demultiplexing process (400 m corresponds to the length of the pulse train in the fiber). Nonetheless, by using a frequency spacing of 100 MHz, the frequency-multiplexed system achieves sufficiently low crosstalk for most applications.

### Summary

In summary, we analyzed the performance capabilities of a coherent φ-OTDR system based on a frequency multiplexed pulse train architecture. The frequency multiplexed pulse train approach allowed us to increase the average power injected into the fiber, enabling lower noise while also providing the diversity required to suppress interference fading and improve the sensor linearity. We experimentally confirmed that increasing the degree of multiplexing improved both the sensor noise and linearity without introducing significant levels of crosstalk. The sensor achieved a THD of − 40 dB, a strain noise of 0.6 $p\varepsilon /\sqrt{\text{Hz}}$ at 10 km with 10 m spatial resolution, and crosstalk below − 40 dB. This system achieves some of the highest performance reported in a Rayleigh-based DAS system with a relatively simple experimental architecture and modest computational requirements. As such, the frequency multiplexed φ-OTDR platform is an attractive candidate for the next generation of high performance DAS systems.

## Supplementary Information

Supplementary Information.

## Data Availability

The data generated during this study are available from the corresponding author upon reasonable request.
