# Supplementary material for: Frequency multiplexed coherent φ-OTDR
Source: Sci Rep. 2021 Sep 9;11:17921. doi: 10.1038/s41598-021-97647-z (PMC8429562; doi:10.1038/s41598-021-97647-z)
Supplement: Supplementary file 1 — Supplementary Information. [file 41598_2021_97647_MOESM1_ESM.pdf]

Frequency multiplexed coherent φ-OTDR

Hannah M. Ogden[1*], Matthew J. Murray[2], Joseph B. Murray[2], Clay Kirkendall[2], Brandon Redding[2]

[1]U.S. Naval Research Laboratory, ASEE Postdoctoral Fellow, 4555 Overlook Ave., SW Washington, DC 20375, USA. *email: hannah.ogden.ctr@nrl.navy.mil

[2]U.S. Naval Research Laboratory, 4555 Overlook Ave., SW Washington, DC 20375, USA

We analyzed the dominant noise sources impacting the self-noise of the frequency multiplexed φ-OTDR sensor presented in this work following the approach described in Ref.'s [1, 2]. In particular, we considered noise introduced by the amplifiers in the form of amplified spontaneous emission (ASE), as well as shot noise, detector noise, and digitizer noise.

The phase noise power spectral density (PSD) in units of rad^2/Hz was calculated as

$$S_\phi = \frac{1}{CNR \cdot PRF \cdot 2 \cdot N_{pulse}} \qquad \text{(Eq. S1)}$$

where CNR is the carrier-to-noise ratio of an individual measurement of one polarization state using a single pulse, PRF is the pulse repetition frequency, N_{pulse} is the number of pulses, and the factor of 2 accounts for measuring both polarization states. The CNR was calculated as

$$CNR = \langle i_{sig}^2 \rangle / \langle i_{noise}^2 \rangle, \qquad \text{(Eq. S2)}$$

where $\langle i_{sig}^2 \rangle$ is the signal power and $\langle i_{noise}^2 \rangle$ is the noise power, both in units of A^2. Assuming heterodyne detection using a local oscillator, the signal power can be calculated as

$$\langle i_{sig}^2 \rangle = 2m \cdot i_{LO} \cdot i_{RBS} \qquad \text{(Eq. S3)}$$

where m is the mixing efficiency (assumed to be 1), i_{LO} is the photocurrent generated by the local oscillator (LO) and i_{RBS} is the photocurrent generated by the Rayleigh backscattered (RBS) light. The LO photocurrent is calculated as $i_{LO} = R_{det} P_{LO}$, where R_{det} is the detector responsivity in units of A/W and P_{LO} is the LO power in units of W reaching the detector. The RBS photocurrent is calculated as $i_{RBS} = R_{det} \cdot \left(\frac{1}{2} \cdot P_0 \cdot r_{RBS} \cdot G_{EDFA-3}\right)$, where P_0 is the peak power injected into the fiber, r_{RBS} is the average reflectance due to Rayleigh backscattering after accounting for the transmission loss in the fiber under test, G_{EDFA-3} is the gain of the 3rd EDFA shown in Fig. 2 which amplifies the RBS light before detection, and the factor of ½ accounts for the polarizing beam splitter inserted before the detectors. Note that the RBS reflectance depends on the pulse duration and we used the specified value of -82 dB/ns for the Corning SMF-28e+ fiber used in this work.

This approach allowed us to calculate the impact of various noise sources on the sensor self-noise. The shot-noise power in units of A^2 was calculated as

$$\langle i_{noise:\,shot}^2 \rangle = 2q\left[i_{LO} + (i_{RBS} \cdot N_{pulse}) + i_{ASE}\right] \cdot (1/\tau) \qquad \text{(Eq. S4)}$$

where q is the charge of an electron in coulombs, i_{ASE} is the photocurrent from the ASE, and τ is the pulse duration. Including the pulse duration accounts for the bandwidth of each measurement so that Eq. S4 provides the noise power in units of A^2. The factor of $i_{RBS} \cdot N_{pulse}$ accounts for the fact that introducing additional pulses will increase the shot noise on each measurement. The photocurrent from the ASE was calculated as $i_{ASE} = \tilde{P}_{ASE} B_0 R_{det}$ where $\tilde{P}_{ASE}$ is the ASE power in units of W/Hz and B_0 is the bandwidth of the ASE in units of Hz (set by the 100 GHz wavelength division multiplexing filter used in this work). The ASE power generated after each EDFA was calculated as $\tilde{P}_{ASE} = (G_{EDFA} - 1) \cdot h\nu \cdot NF$, where G_{EDFA} is the gain of the EDFA, h is Planck's constant, ν is the optical frequency, and NF is the noise factor of the EDFA. The total ASE power was calculated by adding the contributions from each EDFA after accounting for the transmission loss between each EDFA and the photodetector. In this work, we experimentally measured the gain introduced by each EDFA as well as the loss on the path from each EDFA to the detector. The noise power introduced by ASE interfering with the RBS light and the LO was calculated as

$$\langle i_{noise:\,ASE}^2 \rangle = 2\left(P_{LO} + P_{RBS} \cdot N_{pulse}\right)\tilde{P}_{ASE} R_{det}^2 \cdot (1/\tau) \qquad \text{(Eq. S5)}$$

where $P_{RBS} = \frac{1}{2} \cdot P_0 \cdot r_{RBS} \cdot G_{EDFA-3}$ and the factor of N_{pulse} accounted for the impact of additional pulses on the noise of each measurement.

The noise power introduced by the photodetector was calculated as

$$\langle i_{noise:\,det}^2 \rangle = (NEP \cdot R_{det})^2 \cdot (1/\tau). \qquad \text{(Eq. S6)}$$

where NEP is the noise equivalent power of the detector in $W/\sqrt{Hz}$.

The noise power introduced by the digitizer is defined as:

$$\langle i_{noise:\,dig}^2 \rangle = \frac{V_{FS}^2}{2^{(2 \cdot ENOB)}} \cdot \frac{1}{6 \cdot f_s \cdot g_{det}^2} \cdot (1/\tau). \qquad \text{(Eq. S7)}$$

where V_{FS} is the full-scale voltage of the digitizer, $ENOB$ is the effective number of bits, f_s is the digitizer sampling rate, and g_{det} is the detector transimpedance gain in units of V/A which converts the digitizer noise power into equivalent photocurrent noise.

The total predicted noise shown in Fig. 6 and 7 was obtained by summing the noise power from each of these noise sources and adding 3 dB to the phase noise obtained using Eq. S1 to account for calculating the relative phase between RBS light from two reflector regions.

Supplemental References:

1) Kirkendall, C.K. & Dandrige, A. Overview of high performance fibre-optic sensing. *J. Phys. D Appl. Phys.* **37,** R197-R216 (2004).
2) Olsson, N.A., Lightwave Systems With Optical Amplifiers. *J. Light. Technol.* **7,** 1071-1082 (1989).
